# Can bronchial asthma be classified based on the immunological status?

**DOI:** 10.4103/0970-2113.80323

**Published:** 2011

**Authors:** Roopakala Mysore Subrahmanyam, Chandrashekara Srikantaiah, Pushpa Krishna, Chickbllapur Rayappa Wilma Delphine Silvia, Sandeep Thirunavukkarasu, Kusuma Devi, Mohan Rao, Vasantha Kumar

**Affiliations:** *Department of Physiology, M. S. Ramaiah Medical College and Teaching Hospital, Bangalore, Karnataka, India*; 1 *ChanRe Rheumatology and Immunology Center, Bangalore, Karnataka, India*; 2 *Department of Physiology, Kempegowda Institute of Medical Sciences, Bangalore, Karnataka, India*; 3 *Department of Biochemistry, Sapthagiri Institute of Medical Sciences and Research Center, Bangalore, Karnataka, India*; 4 *Department of Physiology, Bangalore Medical College and Research Institute, Bangalore, Karnataka, India*; 5 *Department of Chest Medicine, M. S. Ramaiah Medical College and Teaching Hospital, Bangalore, Karnataka, India*

**Keywords:** Bronchial asthma, cytokines, correlation, immunoglobulin E

## Abstract

**Background:**

Bronchial asthma is a chronic disorder of the airways. Bronchial asthma can be broadly categorized into atopic and nonatopic based on the immunological status, which may be helpful to plan appropriate treatment. In this study, the cytokine profile of the blood was estimated to evaluate the difference between the atopic and the nonatopic asthmatics.

**Aim:**

The aim was to document the differences in cytokine (IL-6, TNF-α, and IFN-γ) imbalance in asthmatics with high serum immunoglobulin E (IgE) levels compared to those with normal serum IgE.

**Materials and Methods:**

Thirty confirmed bronchial asthmatics (17 men, 13 women) were included in this study. The serum levels of IgE, IFN-γ TNF-α, and IL-6 were measured using the enzyme-linked immunosorbent assay technique. They were divided into two groups based on serum IgE: asthmatics with normal serum IgE levels as group A (n = 7) and high IgE levels as group B (n = 23).

**Results:**

: The differences in the mean values of IgE, TNF-α, and IFN-γ were statistically significant between the groups. These values were significantly higher in group B compared to group A. IL-6 was not significantly different between the groups. In group A, IL-6 was negatively correlated with IgE and IFN-γ. IgE and IFN-γ were positively correlated. In group B, IFN-γ and TNF-α showed a high correlation of +0.93.

**Conclusion:**

Two groups of asthmatics stratified according to their serum IgE levels were immunologically different showing different profiles of serum cytokine levels and the relationship between the cytokines.

## INTRODUCTION

Bronchial asthma is a chronic disorder of the airways that is characterized by reversible airflow obstruction, airway inflammation, persistent airway hyperreactivity, and airway remodeling.[1,2] The etiology of asthma is complex and multifactorial. Although profound insights have been made into the pathophysiology of asthma, the exact mechanism inducing and regulating the disease is not fully understood. According to Woodruff *et al*., asthma can be divided into at least two distinctmolecular phenotypes defined by the degree of Th2 inflammation.[3] Simpson *et al*. have shown that the induced sputum eosinophil proportion is a good discriminator for eosinophilic asthma, providing a reproducible definition of a homogenous group. The remaining noneosinophilic subjects are heterogeneous and can be further classified based on the presence of neutrophils.[4] Bronchial asthma is immunologically broadly categorized into intrinsic and extrinsic varieties with more consensus evolving to replace them with terms atopic (allergic) and nonatopic (nonallergic).[5] Though some studies have shown similarities among the two types of asthma[6,7] their clinical characteristics and immunopathogenesis appear significantly different.[8,9] Several markers have been studied to distinguish atopic and nonatopic asthma. But there are limitations in using these parameters for the classification of asthma. However, immunoglobulin E is one of the indicators of atopy and has widely been used to classify asthma.[10] Though defective Th1- and enhanced Th2-type cytokine responses[11,12] have been implicated in the development of the disease, the immunopathology of atopic and nonatopic asthma remains unclear. In order to investigate if atopic asthmatics with higher IgE levels have a cytokine profile comparable to that of nonatopic asthmatics with normal IgE, we estimated the serum levels of IL-6, TNF-α, and IFN-γ levels in these two groups of adult asthmatics. We hypothesized that the two groups of asthmatics differ in the cytokine profile which would further strengthens the concept of classifying them based on IgE levels.

## MATERIALS AND METHODS

Adult asthmatic patients (17 males and 13 females in the age group of 18-60 years), who consented to participate in the study, were enrolled from the Department of Chest Medicine, M. S. Ramaiah Medical Teaching Hospital. Informed consent was taken from the patients. Patients with a history of any significant illness, of smoking, or of being on any drug other than that for asthma and that affects serum cytokine levels were excluded from the study. The study was approved by the institutional ethical committee.

All subjects had their pulmonary function tests (PFTs) done before the blood sample collection. The PFT was done using computerized spirometry (Spirobank G, Wisconsin, USA). FEV1 (Forced expiratory volume in 1 s) was recorded. Asthma patients with reversible airway obstruction, >200 ml or 12% improvement in FEV1 after bronchodilator inhalation, were considered for the study.

Five milliliters of the fasting venous blood sample was collected from each of the subject in the morning. The samples were allowed for clotting, then centrifuged and serum was separated and stored at -20°C until further evaluation. All the samples were analyzed for cytokines and IgE within 3 months of collection.

### IgE assay

Serum IgE levels were measured using the commercially available, approved enzyme-linked immunosorbent assay (ELISA) kit (Genesis Diagnostics, England, UK).

### Cytokine assay

Serum IFN-γ, TNF-α, and IL-6 levels were measured using the DuoSet kit (R&D Systems, Minnesota, USA) by using an ELISA reader (Organon Teknika Microwell System, Reader 230S Germany).

### Statistical analysis

For all parameters, univariate analysis was done to study the mean, standard deviation, and the range. It was noted that the variability of cytokines was high; hence they were logarithmically transformed for analysis. Student *t*-test was applied to compare the mean values between the two groups. A *P*-value less than 0.05 was considered as statistically significant. The correlation coefficient was determined for all possible combinations of cytokines in group A and group B, and the combined resampling technique was used to increase the sample size to see if the correlations were correct. Then the correlation was done taking the cut-off points for the IgE value as 100, 180, 200, and 250 IU/ml in both the groups. Around 500 correlations were done.

## RESULTS

Thirty bronchial asthma patients were recruited for this study. Based on IgE levels, patients were divided into two groups, asthmatics with normal IgE levels (group A) and asthmatics with increased serum IgE levels (group B), the normal IgE level being less than 100 IU/ml. The mean IgE level was 16.72 ± 6.01 IU/ml in group A and 1042.62 ± 143.86 IU/ml in group B. The mean age of group A was 43 ± 12.41 years and that of group B was 38.63 ± 15.5 years. The mean pattern of PFTs in the two groups is shown in Table 1. There was no significant difference in the PFT values between the two groups.

**Table 1:** Differences between group A and group B based on PFT and IgE

Parameters	Group A (*n* = 7)	Group B (*n* = 23)	*P* value
IgE (IU/ml)	16.72 ± 6.01	1042.62 ± 143.86	<0.001
FVC (l)	2.21 ± 1.09	2.58 ± 1.01	0.413
FEV1 (l)	1.60 ± 0.93	1.91 ± 0.83	0.409
PEFR (l/s)	3.42 ± 2.44	3.60 ± 1.54	0.808
PEF 25–75 (l/s)	1.34 ± 0.92	1.77 ± 1.21	0.393

IgE: Immunoglobulin E; FVC: Forced vital capacity; FEV1: Forced expiratory volume in 1 s; PEFR: Peak expiratory flow rate; PEF 25–75 = Peak expiratory flow between 25% and 75% of the vital capacity.

The mean pattern of cytokines in the two groups is shown in Table 2. The difference in the mean values of IgE, TNF-α, and IFN-γ were statistically significant between group A and B (P<0.001, *P* = 0.02, and *P* = 0.02, respectively). IL-6 was not significantly different between the groups (*P* = 0.19; Table 2). In Table 2, the mean and standard deviations are presented in the original units whereas the P-value corresponds to the comparison of log-transformed data. The reason for log transformation was to reduce the variability. The results of correlation analysis are given in Table 3. In group A, IL-6 negatively correlated with IgE (–0.62) and IFN-γ (–0.52). IgE and IFN-γ were positively correlated (+0.78). In group B, IFN-γ and TNF-α showed a high correlation of +0.93; however, IgE is independent of both IFN-γ and TNF-α. Resampling analysis showed that 86% of the samples showed a good correlation (0.75-1) between IFN-γ and IgE in group A [Table 4] with a cut-off value of IgE as 100 IU/ml. In group B, IFN-γ and TNF-α showed a high correlation of +0.93 in 94% of the samples.

**Table 2:** Correlation between group A and group B with IgE, TNF-α, IL-6, and IFN-γ

Study parameters	Group A (Mean ± SD)	Group B (Mean ± SD)	*P* value* (two-tailed)	*P* value* (one-tailed)
IgE (IU/ml)	16.72 ± 6.01	1042.62 ± 143.86	<0.001	<0.001
TNF-α (pg/ml)	5.16 ± 4.40	49.88 ± 138.24	0.02	0.01
IL-6 (pg/ml)	0.81 ± 0.47	4.75 ± 13.67	0.19	0.09
IFN-γ (pg/ml)	15.30 ± 8.95	68.94 ± 101.61	0.02	0.01

P<0.05 statistically significant

**Table 3:** Correlation of TNF- α, IL-6, IFN-γ, and IgE between group A and group B

Variables	Group A	Group B	Group B
IFN-γ vs. TNF-α	0.06	0.93	0.93
IFN-γ vs. IL-6	–0.52	–0.06	–0.03
TNF-α vs. IL-6	–0.24	0	0.02
TNF-α vs. IgE	0.43	0.18	0.25
IgE vs. IFN-γ	0.78	0.12	0.27
IgE vs. IL-6	–0.62	–0.02	0.09

**Table 4:** Percentage of samples showing absolute correlation values between 0.75 and 1 (good correlation) for group A

IgE cut-off	≥100 IU/ml (%)	≥180 IU/ml (%)	≥200 IU/ml (%)	≥250 IU/ml (%)
IFN-γ vs. IL-6	20	24	27	21
IFN-γ vs. IgE	86	58	24	25
IgE vs. IL-6	39	56	20	24

## DISCUSSION

In this study, we have divided adult bronchial asthma patients into two groups based on serum IgE levels (the cut-off being 100 IU/ml) and evaluated their pulmonary functions and the serum cytokines IL-6, TNF-α, and IFN-γ levels. We have considered total IgE as it is proved that asthma is associated with increased levels of total IgE, even in subjects negative for specific IgE to common aeroallergens.[13] The difference in the mean values of IgE, TNF-a, and IFN-γ was statistically significant between the groups (P<0.05). Furthermore, these values were significantly higher in group B compared to group A (*P* < 0.05). The mean values of IFN-γ and TNF-α in group B were 4 and 10 times the mean of group A, respectively. IL-6 was not statistically significantly different between the groups; however, the mean value in group B was six times the mean value in group A [Table 2]. These results are in accordance with the proposed hypothesis that the two groups of asthmatics classified based on serum IgE levels show significant differences in cytokine levels. There was no significant change in the pulmonary function tests between the two groups. PFTs and therefore the severity of the disease were the same in both the groups.

We could find that there was a significant difference in the relationship as well the serum levels of TNF-α and IFN-γ in patients with high serum IgE (group B) and low IgE (group A). The cytokines, both TNF-α and IFN-γ, were significantly higher in the group of patients with higher serum IgE and this difference could be seen at 175 IU/ml of serum IgE. Looking at this pattern, it can be concluded that IFN-γ and TNF-α secreted by Th1 cells were higher in group B with high IgE levels and IgE levels had no influence on the levels of these cytokines. In group B (with high IgE), TNF-α and IFN-γ levels were directly proportional, while these levels were not influenced by serum IgE levels.

In patients with low levels of serum IgE (group A), levels of IFN-γ and TNF-α were low and these levels had no influence on each other. In this group, IFN-γ levels were positively correlating to serum IgE, while serum IgE and IFN-γ had a negative effect on IL-6. This suggests a differential cytokine relationship between the two groups with respect to IgE. Elevated levels of Th1 cytokines TNF-α and IFN-γ are not surprising since there are adequate previous publications.[12,14] This is attributed to allergen-specific Th1 response, which can exacerbate bronchial hyperresponsiveness. A higher concentration of Th1 cytokines was found in patients with higher IgE supporting the observation that the allergen specific Th1 response may be an active pathway in group B.[15] IFN-γ has been demonstrated to increase the expression of low-affinity IgE receptors on monocytes. The activation of these receptors by IgE immune complexes stimulates the release of inflammatory mediators such as leukotrienes, platelet-activating factor, IL-1b, and TNF-α.[16] The release of the TNF-α is also enhanced by the IgE-dependent stimulation of alveolar macrophages.[16] Thus, the influence of IFN-γ on the level of TNF-α was obvious in our study, but the level was not influenced by serum IgE levels. In addition, several studies have shown an increase in serum levels of IFN-γ in asthma and in bronchoalveolar lavage fluids in asthma.[17] No relationship was observed between the severity and chronicity of asthma with either TNF-α or IFN-γ level. Surprisingly, in contrast to the data which report that Th2-mediated inflammation is dominant in an allergen-induced asthma, several papers have shown that the addition of allergen-specific Th1 cells can exacerbate airway inflammation. A physiological role of IgE in the airway is required for balancing both Th1 and Th2 responses to prevent immoderate inflammation dominated by either.[18]

One of the drawbacks of our study was the number of patients in either group. The sample size of 7 in group A is small as compared to the sample size of group B that was 23. The consistency of correlation coefficients determined using these data was explored using the resampling method. Furthermore, it was also determined if the IgE value of 100 could be used as an optimum threshold value to divide the subjects. Based on the results, it can be said that a consistent correlation is seen in group B strengthening the theory of higher Th1 cytokines in atopic asthmatics. But in group A, the positive correlation between IFN-γ and IgE is consistent as compared to other relations. We could find that there was a significant difference in the relationship of TNF-a and IFN-γ in patients with high serum IgE and low IgE. The cytokines, both TNF-α and IFN-γ were significantly higher in the group of patients with higher serum IgE and this difference could be seen up to 175 IU/ml of serum IgE.

The available epidemiological evidence suggests that the population-based proportion of asthma cases that are attributable to atopy is usually less than one-half.[19] Higher estimates (up to two-thirds) can be obtained by using very low cut-off levels of total serum IgE, but these should be interpreted with caution since such a definition of atopy has a limited practical use, and these associations may not always be causal. The limitation of this study is that the status of the fungal sensitization in the patients, specifically the work-up for allergic bronchopulmonary aspergillosis (ABPA), was not carried out in asthmatics especially those found to have IgE levels more than 1000 IU/ml. A prospective study with a larger number of patients from different clinical backgrounds should help to further reinforce this observation.

## CONCLUSION

Serum levels of TNF-α and IFN-γ are higher in atopic asthmatics compared to nonatopic asthmatics and the two groups of asthmatics stratified according to their serum IgE levels are immunologically different showing different profiles of serum cytokine levels and their relationship. Understanding this relationship should help to analyze these groups and to plan appropriate treatment.
